# Glucosamine and N-acetyl glucosamine as new CEST MRI agents for molecular imaging of tumors

**DOI:** 10.1038/srep32648

**Published:** 2016-09-07

**Authors:** Michal Rivlin, Gil Navon

**Affiliations:** 1 School of Chemistry, Faculty of Exact Sciences, Tel Aviv University, Tel Aviv, Israel.

## Abstract

The efficacy of glucosamine (GlcN) and N-acetyl glucosamine (GlcNAc) as agents for chemical exchange saturation transfer (CEST) magnetic resonance molecular imaging of tumors is demonstrated. Both agents reflect the metabolic activity and malignancy of the tumors. The method was tested in two types of tumors implanted orthotopically in mice: 4T_1_ (mouse mammary cancer cells) and MCF7 (human mammary cancer cells). 4T_1_ is a more aggressive type of tumor than MCF7 and exhibited a larger CEST effect. Two methods of administration of the agents, intravenous (IV) and oral (PO), gave similar results. The CEST MRI observation of lung metastasis was confirmed by histology. The potential of the clinical application of CEST MRI with these agents for cancer diagnosis is strengthened by their lack of toxicity as can be indicated from their wide use as food supplements.

Early detection of tumors, discrimination between benign and malignant tumors, and monitoring tumor response to therapy are major challenges in diagnostic imaging^1^. One of the fundamental differences between normal cells and rapidly proliferating cancer cells is the altered metabolism of the latter. Cancer cells regulate their consumption and processing of glucoses, fats, amino acids and other energy sources to satisfy the demands of continuous proliferation. These altered metabolic processes can be a fundamental driver of tumor growth. Positron emission tomography (PET) with the contrast agent 2-(^18^F)-fluoro-2-deoxy-D-glucose (FDG)^2^ is targeting on one of its characteristic features: glucose uptake. It is usually performed together with computer tomography (PET/CT) – a combination that has more than 90% sensitivity and specificity for the detection of metastases of most epithelial cancers^3^. Both PET and CT techniques employ ionizing radiation for detection, making their repetitive use problematic.

Glucosamine (2-amino-2-deoxy-D-glucose, GlcN) and N-acetyl glucosamine (GlcNAc) are amino monosaccharides that are components of glycosaminoglycans, which constitute a major part of the matrix of all connective tissues. The entry of GlcN into cells is stimulated by insulin and involves the glucose-transporter system. When free GlcN enters cells, its downstream metabolism is significantly limited to its phosphorylation (to glucosamine-6-phosphate)^4^,^5^,^6^. Enhanced accumulation of GlcN was demonstrated for cultured Novikoff rat hepatoma cells^7^. Such accumulation is expected in all tumors that overexpress the glucose transporters GLUT1 and GLUT2. While GLUT1 has similar affinity for glucose and GlcN, GLUT2 has about 20-fold higher affinity for GlcN than for glucose^8^. GLUT1 and GLUT2 are expressed in a variety of tumors including breast invasive ductal carcinoma, and not in normal breast tissue^9^.

We propose a new modality to detect tumors based on GlcN and GlcNAc chemical exchange saturation transfer (CEST) MRI. This technique enables indirect imaging of metabolites *in vivo* via magnetization transfer between exchangeable protons of their functional groups and the water protons^10^,^11^,^12^,^13^,^14^. Saturation of the exchangeable protons by selective radio-frequency (RF) irradiation attenuates the bulk water signal, leaving the intensity of the image commensurate with the concentration of the metabolite. GlcN has four hydroxyl protons and two amino protons and GlcNAc has four hydroxyl protons and one amide proton: all are potential candidates for CEST, but their relative contribution depends on the proton exchange rate. Further CEST signals can be obtained from the exchangeable protons of GlcN^4^ or GlcNAc^15^,^16^ metabolic products.

CEST MRI using D-glucose, 2-deoxy-D-glucose (2-DG), FDG, and 3-O-methy-D-glucose (3OMG) was recently shown to be useful for imaging the viable parts of tumors implanted in mice^17^,^18^,^19^,^20^,^21^. Relative to the other CEST agents, D-glucose showed inferior sensitivity and shorter duration of the effect^19^,^20^ owing to its rapid metabolism in the tumors. Both FDG and 2-DG enter the tumor cells through GLUT family members where they and their phosphorylated products accumulate, but they may be toxic at high concentrations. The non-metabolizable 3OMG may be a better CEST MRI alternative^21^, but detailed studies of its toxicity are lacking. In the present work the non-toxic GlcN and GlcNAc are tested as potential candidates for tumor imaging by CEST MRI.

## Results

### *In vitro*

**Figure 1:**
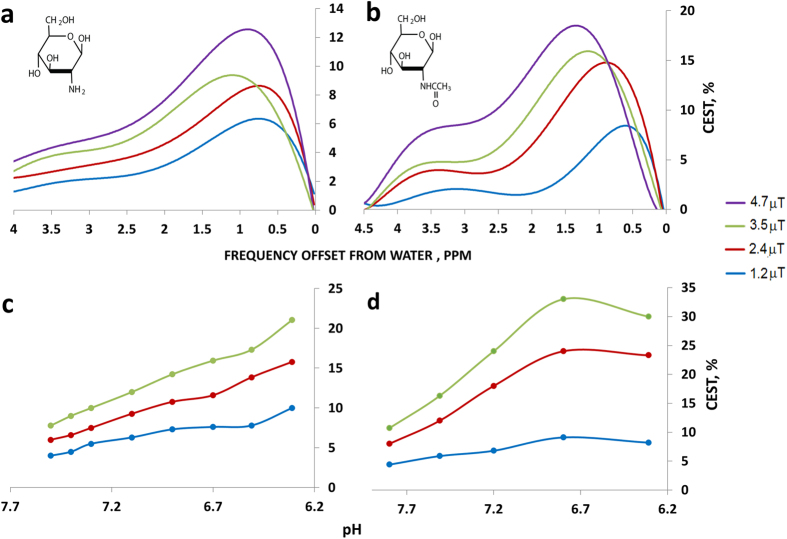
Radiofrequency (RF) saturation field (B_1_) and pH dependencies of CEST contrast in 20 mM GlcN sulfate and GlcNAc solutions (containing 10 mM PBS and 10% D_2_O) at a temperature of 37 °C at 11.7 T. (**a**,**b**) CEST plots as a function of the offset at pH = 7.4. (**c**,**d)** The pH dependencies of the CEST of GlcN sulfate and GlcNAc solutions, respectively, at an offset of 1.2 ppm from the water signal.

*In vitro* CEST NMR experiments were performed to test the ability of the method to detect millimolar concentrations of GlcN and GlcNAc under physiological conditions. Figure 1 presents the % CEST plots at different RF saturation fields (B_1_) for solutions of 20 mM GlcN and GlcNAc at 37 °C and pH 7.4, corresponding to the intracellular conditions. Both GlcN and GlcNAc showed a significant CEST effect around a frequency offset of 1.2 ppm from water that originated from the hydroxyl groups connected to carbons numbers 3 and 4. At this offset, and for B_1_ of 2.4 μT, the CEST effects for the 20 mM solutions of GlcN and GlcNAc at pH 7.4 amounted to 7% and 14% respectively. Considering the proton concentration in water, these results correspond to an enhancement by factors of 350 and 700, respectively, over the direct detection of the metabolites. As can be seen from the pH dependence shown in Fig. 1c,d, the CEST effect is significantly larger at lower pH values, which may be pertinent to the extracellular microenvironment of tumors known to be slightly acidic.

GlcN and GlcNAc showed significant CEST effects through the amino protons at a frequency offset of ~3 ppm and the amido protons at frequency offset of ~3.5 ppm (Fig. 1a,b). However these CEST effects were lower than that observed at frequency offset of 1.2 ppm.

### *In vivo*

**Figure 2:**
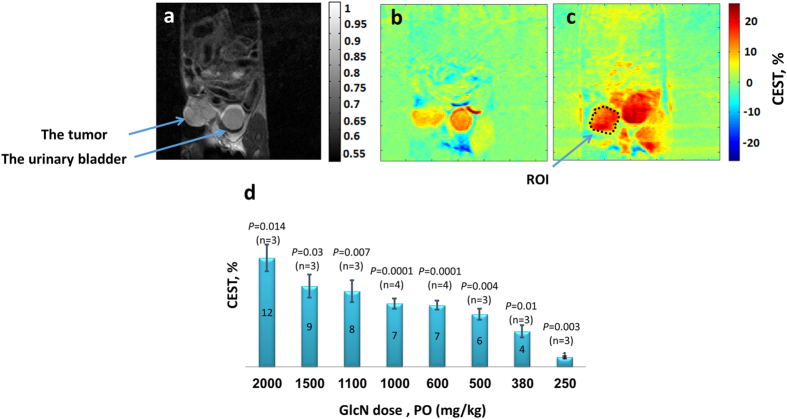
GlcN CEST MRI results for mice bearing 4T_1_ breast tumors at B_0_ = 7 T. (**a)** A T_2_ weighted image for a mouse bearing a 4T_1_ breast tumor (before administration of the agent). (**b**) A CEST image before administration of the agent; at frequency offset of 1.2 ppm, B_1_ = 2.4 μT, 10.4% CEST was obtained in the tumor. (**c**) A CEST image 48 min after PO treatment with GlcN, 1.1 g/kg; at a frequency offset of 1.2 ppm, B_1_ = 2.4 μT, 20.2% CEST was obtained in the tumor. The CEST calculation was made for the marked ROI. (**d**) Bar graph showing mean ± standard deviation (and *P* values) of GlcN CEST contrast (one hour after GlcN administration) for 4T_1_ tumors.

**Figure 3:**
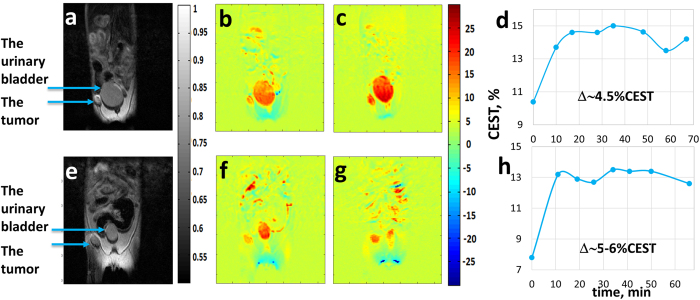
Images of CEST MRI kinetic measurements in mice bearing MCF7 tumors (human cancer cells) at different times following IV (**a–d**) and PO (**e–h**) administration of GlcN, 1.0 g/kg (B_1_ = 2.4 μT, B_0_ = 7 T). (**a,e**) A T_2_-weighted image before administration of the agent (the conventional image). (**b,f**) A CEST image before administration of the agent (10.4% and 7.8% CEST in the whole tumor, respectively). (**c,g**) A CEST image 48 and 41 mins after administration of the agent (14.6% and 13.4% CEST in the whole tumor, respectively). (**d,h**) The time series of the % CEST achieved in the tumor.

**Figure 4:**
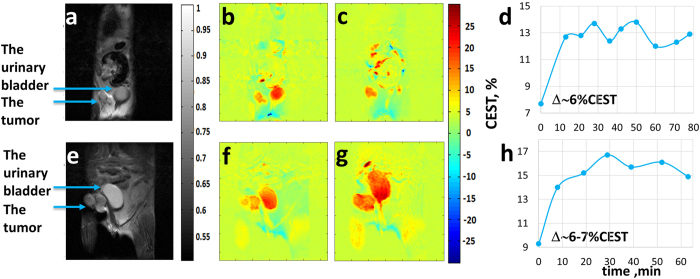
Images of CEST MRI kinetic measurements in mice bearing 4T_1_ tumors at different times following PO **(a–d)** and IV **(e–h)** administration of GlcNAc, 1.0 and 1.1 g/kg, respectively. (B_1_ = 2.4 μT, B_0_ = 7 T). (**a,e**) A T_2_-weighted image before administration of the agent (the conventional image). (**b,f**) A CEST image before the administration of the agent (7.7% and 9.3% CEST in the whole tumor, respectively). (**c,g**) A CEST image 50 and 52 min after administration of the agent (13.8% and 16.1% CEST in the whole tumor, respectively). (**d,h**) The time series of the % CEST achieved in the tumor.

**Figure 5:**
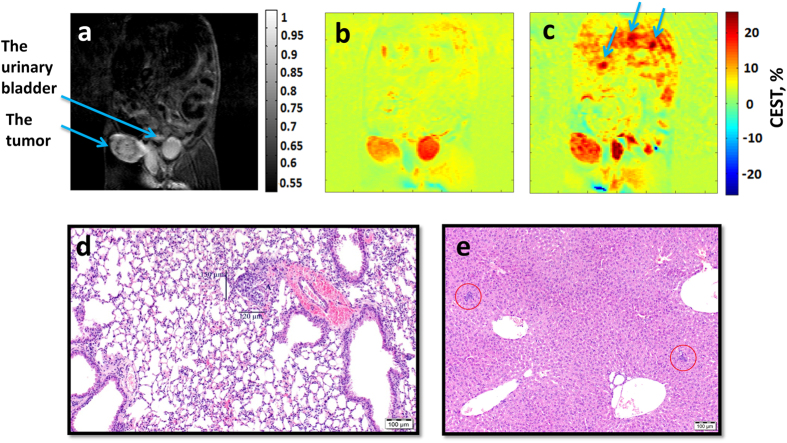
T_2_-weighted (**a**) and control CEST MRI (**b**) images before treatment vs. GlcN CEST MRI contrast (**c**) for a mouse bearing 4T_1_ tumor (treated with 1 g/kg GlcN, PO). (**d**) Hematoxylin and eosin stain histology of the right lung. (**e**) Hematoxylin and eosin stain histology of the liver.

The *in vivo* experiments were carried out on two widely-used breast cancer xenograft models, 4T_1_ tumors (aggressive mouse breast cancer cells) and MCF7 tumors (human breast cancer cells), implanted in 8-week-old female BALB/C and SCID/ICR mice, respectively. Two methods of administration of the CEST agents were tested: intra-venous through the tail vein (IV), and per-os by oral gavage (PO). The tumors in both 4T_1_ and MCF7 models showed significant CEST effects lasting for more than an hour following administration of GlcN or GlcNAc (Figs 2, 3, 4 and 5).

In the 4T_1_ model, PO administration of 0.38–2.0 g/kg GlcN increased the CEST signals of the tumors by 4–12% relative to the baseline (Fig. 2d). A one-way ANOVA was used to test for differences in the obtained % CEST among GlcN doses, showing statistically significant differences (*P* < 0.0001); Tukey’s *post hoc* test showed that the differences are between 5–6 groups of doses. The relatively large CEST signal at baseline, 9.9 ± 0.6% (mean ± standard deviation (STD); *n* = 26) is probably due to the presence of other metabolites, such as the glycosaminoglycans^22^,^23^ and poly-sialic acid residues of mucoproteins known to be abundant in many tumors^24^,^25^. However, following GlcN administration, there was a substantial increase in the CEST effect; for example, GlcN at a dose of 1.1 g/kg raised the CEST effect from a baseline of 10.4% to 20.2%, a 9.8% increase (Fig. 2c). IV administration of GlcN to mice carrying 4T_1_ tumors showed the same effect as those for PO administration.

CEST MRI experiments were performed in 6 SCID-ICR mice carrying the human MCF7 cell line treated with a dose of 1.0 g/kg GlcN, 3 administered by PO and 3 by IV (Examples are shown in Fig. 3). The CEST signals of MCF7 tumors increased by 5.5 ± 1.1% (*n* = 3, *P* = 0.014) for the PO experiments and by 5.4 ± 0.8% (*n* = 3, *P* = 0.007) for the IV experiments. Signal changes were also seen in the urinary bladder, as expected, and no significant changes were observed in other organs. The % CEST obtained in the tumors rose rapidly during the first 10 mins after treatment (Fig. 3d,h) by both modes of delivery, followed by steady state persistence of the CEST effect for more than an hour. It is interesting to note that the CEST effects for the MCF7 tumors were consistently lower than those for the 4T_1_ tumors. This is compatible with the less aggressive nature of the MCF7 tumors as seen from the time of growth of about 30 days compared to 10 days for the 4T_1_ tumors.

CEST MRI experiments were performed in 10 Balb/C mice carrying 4T_1_ tumors treated with a dose of 1 g/kg GlcNAc, 5 by PO and 5 by IV (Examples are shown in Fig. 4). The CEST percent of 4T_1_ tumors increased by 5.9 ± 0.7% (*n* = 5, *p* < 0.0001) for the PO experiments and by 6.1 ± 0.9% (*n* = 5, *p* < 0.0001) for the IV experiments. The tumor in Fig. 4a occupies two areas and that in Fig. 4e three areas. In both cases the animal was treated with GlcNAc (1.0 g/kg PO and 1.1 g/kg IV, respectively). The CEST effect was observed in the whole tumor (Fig. 4c,g), thus most of the tumors appear to be active.

An example showing the detection of metastasis by CEST MRI following the administration of 1.0 g/kg GlcN is shown in Fig. 5. This experiment was done in Balb/C mice bearing 4T_1_ tumor, in their primary advanced stage (~14 days after injection of cells). These tumors are known to metastasize from the primary tumor in the mammary gland to multiple distant sites including lymph nodes, blood, liver and lungs^26^. The GlcN CEST MRI experiment points to three areas suspected for metastasis in the liver and in the lower portion of the right lung (as indicated by arrows in Fig. 5c). The % CEST at these points was 12–13% above the baseline (Fig. 5b). The presence of metastasis was confirmed by histology (Fig. 5d,e).

## Discussion

The results of this study demonstrate the ability of CEST MRI using the amino monosaccharides GlcN and GlcNAc to serve as contrast agents to detect tumors. The validity of the method was established by the findings that both PO and IV administration of GlcN or GlcNAc to mice bearing 4T_1_ and MCF7 implanted breast tumors significantly enhanced the CEST MRI contrast of the tumors. The improved CEST MRI images started within a few minutes of GlcN or GlcNAc administration and persisted for over an hour (Figs 3d,h and 4d,h); the mice appeared to be unaffected by the treatment.

The larger CEST effect obtained for the more aggressive 4T_1_ tumors points to the technique as a possible indicator of the aggressiveness of the tumor, something that cannot be obtained by standard MRI experiments. The *in vitro* CEST signal produced in solutions of GlcNAc was higher than that produced in GlcN solutions (Fig. 1). On the other hand, the CEST contrast observed for 4T_1_ tumors treated with GlcN was slightly higher than that observed with GlcNAc (Fig. 2b,c vs. Fig. 4b,c). This may be due to greater accumulation of GlcN in the tumor. It should be noted that not only the accumulated GlcN and GlcNAc are responsible for the observed CEST effects but some of their metabolic products^4^,^15^,^16^ may also contribute to the effect. This phenomenon is presently under investigation.

A major advantage of these agents is their lack of toxicity, which makes them suitable for human clinical diagnosis. Indeed, they are being used in the treatment of osteoarthritis^27^,^28^,^29^,^30^ and inflammatory bowel disease^31^. The reason for their use for osteoarthritis is the fact that GlcN and GlcNAc are precursors for glycosaminoglycans (GAGs) molecules, that are major components of joint cartilage. In addition to the clinical studies leading to the conclusion that GlcN and GlcNAc have little if any potential for harm^31^,^32^,^33^, human breast milk contains up to 1,500 milligrams of GlcNAc per liter^34^, which means that a breast-feeding newborn may consume as much as 900 mg of GlcNAc a day.

Oral administration of GlcN at very high doses (5–15 g/kg body weight) was found to be well tolerated in humans^4^. As GlcN and GlcNAc have been widely prescribed as a nutritional supplement for therapeutic usage, it should be possible to perform oral administration in the clinic. Indeed, GlcN is usually taken orally and in humans 90% is absorbed^35^,^36^.

Significant CEST MRI contrast was achieved in our study when the animal was treated with a dose of 380 mg/kg GlcN, PO (Fig. 2d). The equivalent human oral dose would be about 2 g per 70 kg (calculation based on the ratio of mouse to human surface area). Human plasma pharmacokinetics showed that when 3 g of glucosamine sulphate were administered orally to healthy volunteers, the GlcN plasma concentration reached its maximum of about 14 mM at about 3.5 hours, and declined to half the maximum at about 8 hours from administration^37^. This should give ample time to perform the CEST MRI examination.

An advantage of both GlcN and GlcNAc over D-glucose is their suitability for diabetic patients; some studies suggest that they have no significant effects on insulin or blood glucose values^38^,^39^,^40^,^41^,^42^,^43^,^44^.

Our results were performed using cw irradiation at 1.2 ppm from the water peak on a 7 T MRI scanner. Human 7 T MRI scanners are not used in clinical practice at the present time. The proximity of the irradiation frequency to the water peak may impede the application of our technique using MRI scanners with lower fields, such as 3 T. Further development of the CEST technique is required to cope with this problem. The use of the amine and amide proton peaks of GlcN and GlcNAc that occur at 3.0 and 3.5 ppm from the water peak should also be explored; while they give smaller CEST effect than the peak at 1.2 ppm, their effect at lower field strengths is not known.

In summary, both GlcN and GlcNAc have demonstrated good tumor targeting properties with *in vivo* CEST MRI. This new modality offers good resolution, and sensitivity sufficient to detect metabolic changes. The method, which requires no additional equipment, can replace PET/CT or PET/MRI as a standard imaging modality for the detection of tumors and their metastases as well as their response to therapy. The method is especially suitable for diabetics as GlcN and GlcNAc do not appear to affect blood glucose levels or insulin sensitivity. The advantage of CEST MRI of glucose and its analogs over other MRI modalities is that it reflects metabolic changes that often occur with no apparent morphological changes, enabling clinicians to stage the disease at diagnosis.

## Methods

### Chemicals

GlcN hydrochloride and GlcNAc were obtained from Sigma-Aldrich, Israel. GlcN sulfate was obtained from Carbosynth Ltd, (Compton, UK).

### *In vitro* studies

Solutions of GlcN sulfate and GlcNAc were prepared in 10 mM phosphate-buffered saline (PBS) containing 10% D_2_O for NMR field/frequency locking.

#### NMR Spectroscopy

NMR spectra were acquired on a Bruker DRX spectrometer (Germany), equipped with a 11.7 T wide-bore vertical magnet, operating at an RF of 500 MHz for ^1^H detection. The probe temperature was set to 37 °C for all experiments. The spectrometer was de-tuned to avoid radiation damping effects. The 45° pulse duration was ~16 μs, and the acquisition time and relaxation delay were 2 s and 8 s, respectively. Spectral width was 7500 Hz, data size 16 K, number of scans = 8, and a saturation pulse of 3 s was employed at a series of frequencies (Ω) in the range of −5 to +5 ppm relative to the water signal (0 ppm). Several RF saturation fields (B_1_) in the range of 1–6 μT were used. The data were recorded and processed using TOPSPIN 2.1 software (Bruker).

The CEST was measured by magnetization transfer asymmetry:


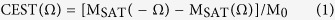


### *In Vivo* Experiments

#### Tumor-bearing animals

##### Cell culture

4T_1_ (mouse mammary cancer cells) and MCF7 (human mammary cancer cells) were obtained from American Type Culture Collection (ATCC). Cells were cultured in high glucose DMEM supplemented with 10% PBS, 100 μg/ml penicillin, 100 U/ml streptomycin, 12.5 U/ml nystatin and 2 mM L-glutamine (Sigma, Israel) at 37 °C in a humidified atmosphere containing 5% CO_2_.

### Orthotopic mammary fat pad implantation

BALB/C or severe combined immune deficiency (SCID-ICR) female mice were purchased and housed in the breeding facility of Tel Aviv University. Orthotopic xenograft tumors were induced in the mice by injecting 4T_1_ cells or human MCF7 cells (10^6^/100 μl cells) into the lower left mammary gland of 6–8-week-old mice (Envigo, Israel), weighing 17–22 grams, using a 27-gauge needle. The tumors were allowed to grow for 10–14 days (4T_1_) or 30 days (MCF7), reaching an average tumor volume of 5 ± 2 mM^3^. Mice were imaged by CEST MRI on week 2 (4T_1_ Model) or 3–5 (MCF7 Model) post implantation. All experiments with animal models were carried out in compliance with the principles of the Israel National Research Council (NRC) and were approved by the Tel Aviv University Institutional Animal Care and Use Committee (IACUC) (#M-15–057, 01-16-005).

### CEST MRI protocol

A Bruker 7 Tesla (T) BioSpec scanner with 30 cm bore size was used to scan implanted xenograft mammary tumors of mice before and after administration (IV or PO) of GlcN hydrochloride or GlcNAc (dissolved in saline, pH 7.4). Mice bearing 4T_1_ or MCF7 tumors, with an average tumor volume of 5 ± 2 mm^3^, were fasted overnight with water access, anesthetized with isoflurane (1–2%), and scanned with a volume coil that was used for RF transmission (without surface coil). Mouse body temperature was maintained at 37 °C.

T_2_ RARE anatomical images (RARE factor-8, TR-3000 ms, TE-11.7 ms) were acquired to identify the slice presenting the maximum tumor size. The CEST images were generated as follows: a series of T_2_ RARE images (RARE factor-1, TR-3000 ms, TE-11.7 ms) was collected from a single 1 mm coronal slice of the abdominal area (acquisition matrix before zero filling 128 × 64, field of view of 40 × 40 mm^2^) after a 2 s presaturation pulse of B_1_ = 2.4 μT at the hydroxyl proton frequency offset of ±1.2 ppm from the water signal. CEST images were acquired before and for 1–2 hours after GlcN or GlcNAc administration. The mean intensities in the selected region of interest (ROI) of the tumor were used for the % CEST plot. The reported statistical analyses in all experiments were given as standard deviations (±SD).

No corrections for the B_0_ inhomogeneity were used in the present work because no significant change was observed after such corrections in our previous works. The line width of the water peak was approximately 30 Hz.

### Histology

Immediately after the CEST MRI experiment, the tumor, lungs, kidney, ovaries, uterus and liver were excised and fixed in 4% formalin solution. Pathology analysis was performed by Patho-Lab (Rehovot, Israel).

### Statistical Analysis

The results were evaluated using the statistical software SPSS 23.0. All data were expressed as mean ± standard deviation (STD). One-way analysis of variance for multiple comparisons with Tukey post-hoc test were used to determine statistical significance, which was defined by a *P* value < 0.05.

## Additional Information

**How to cite this article**: Rivlin, M. and Navon, G. Glucosamine and N-acetyl glucosamine as new CEST MRI agents for molecular imaging of tumors. *Sci. Rep.* **6**, 32648; doi: 10.1038/srep32648 (2016).
